# Polish Consumers’ Attachment to Meat: Food and Plant-Based Meat Alternative Choices

**DOI:** 10.3390/nu17081332

**Published:** 2025-04-11

**Authors:** Małgorzata Kosicka-Gębska, Marzena Jeżewska-Zychowicz, Marta Sajdakowska, Jerzy Gębski, Krystyna Gutkowska

**Affiliations:** Institute of Human Nutrition Sciences, Warsaw University of Life Sciences (SGGW-WULS), Nowoursynowska 159C, 02-776 Warsaw, Poland; marzena_jezewska_zychowicz@sggw.edu.pl (M.J.-Z.); marta_sajdakowska@sggw.edu.pl (M.S.); jerzy_gebski@sggw.edu.pl (J.G.); krystyna_gutkowska@sggw.edu.pl (K.G.)

**Keywords:** meat, meat attachment, plant-based meat alternatives

## Abstract

**Background/Objectives:** Poland has remained one of the leaders in meat consumption in Europe for years. This study aimed to determine the relationship between Polish consumers’ meat attachment and willingness to change their consumption habits to plant-based foods and meat, using self-assessment, including attitudes toward health, naturalness of food, product novelty, and willingness to seek information about these products. In addition, the relationship between the meat attachment of the surveyed individuals consuming plant-based meat alternatives and their attitudes towards these products was assessed. **Methods:** A representative survey with 1003 respondents was conducted using the CAWI (computer-assisted web interview) method. Four consumer clusters were identified using the k-means method: Meat lovers, Meat neutral, Meat attached, and Meat opponents. **Results:** The Meat lovers cluster was characterized by dominant values for entitlement and hedonism and the least willingness to consume plant-based products in the coming year. It was represented by those who care about their health and like product novelty while lacking familiarity with and willingness to try plant-based meat alternatives. The Meat opponents cluster was distinguished by having the highest readiness for eating plant-based products. These individuals valued the naturalness of food, disliked buying new food products, and were familiar with plant-based meat alternatives but consumed them occasionally. Furthermore, cluster membership differed after considering gender, education level, and place of residence. **Conclusions:** The findings indicate the need for educational and marketing activities to raise awareness of the health and environmental benefits resulting from reducing meat consumption to decrease meat attachment.

## 1. Introduction

The impact of meat on health and the environment is the subject of research and much controversy [1,2]. It is classified as a product of high nutritional value [3,4,5,6]. It is an essential part of a balanced diet, which is necessary for optimal human growth and development [6]. On the other hand, the fat content of meat is a health barrier [7]. There is also concern about the proven impact of meat consumption on the increased risk of cancer, cardiovascular disease, obesity, and type 2 diabetes [8,9,10].

In addition, consumers are increasingly aware of the negative impact of animal farming on the environment, including water consumption, freshwater pollution by sewage, greenhouse gas emissions, and deforestation [11]. Ethical issues are also considered [12], as well as animal welfare [13,14]. To avoid a global environmental catastrophe, meat consumption would have to decrease by an average of 75% [15], which, given the projected increase in demand due to world population growth, seems to be a huge challenge [16].

In this context, there is a need to search for new sources of food and, thus, nutrients necessary for the proper functioning of the human body. Developing new ways of producing food is a challenge for the modern food systems of the world [17,18]. Knowledge of the negative impacts of meat production and consumption is resulting in new trends in consumer behavior towards this category of products. Specifically, it influences the reduction in or abandonment of their consumption [19], which helps to popularize the idea of a balanced diet [20,21]. In recent years, many people have adopted meat-free diets, such as vegetarian, vegan, or flexitarian [22]. These trends are also reinforced by global and national dietary recommendations [23,24] advocating a reduction in the consumption of food of animal origin in favor of food of plant origin [12,25], especially soybeans, beans, lentils, chickpeas, and peas, as well as cereal products, nuts, and seeds [26,27]. The inclusion of alternative protein sources in the diet is also promoted, including insects, fungi, algae, yeast, and cultured meat [28]. However, the lack of acceptance of this type of protein source in some cultures and concerns about health safety resulting from consuming food containing them limit the increase in the share of this type of food in the diet [29].

In recent years, many food manufacturers have been introducing foods with similar functional characteristics to meat products (plant-based meat alternatives—PBMA), which may favor a reduction in meat consumption. These products are designed to satisfy consumer expectations not only in terms of taste and smell but also in terms of nutritional properties. These include pastries, burgers, minced vegetable meat, sausages, meatballs, bacon, hot dogs, and many more [28]. This new generation of products is designed to imitate meat using structuring processes (e.g., extrusion) and ingredients derived from legumes, grains, and oilseeds to simulate the taste, texture, aroma, appearance, and culinary sensations of meat [30].

The studies conducted so far have shown that for consumers, the most essential factors in choosing food, including meat, are the individual’s cognitive factors related to food. These include attitude, preference, knowledge and skills, personal identity, and anticipated consequences [31]. Research has shown that people’s willingness to reduce their meat intake or their willingness to increase their PBMA intake is probably related to their meat attachment (MA) [32,33,34,35]. MA refers to the positive association with meat consumption. To measure MA, Graça et al. [36] developed the Meat Attachment Questionnaire (MAQ), which includes four dimensions: hedonism (the perception of meat as a source of pleasure), affinity (an affinity with meat consumption or perhaps a connection with meat consumption), sense of entitlement (the feeling of being entitled to meat consumption), and dependence (the feeling of being addicted to meat consumption). The interaction of these dimensions shapes the degree of an individual’s attachment to meat consumption, so consumers who are firmly attached to meat consume more meat and perceive the intention to follow a plant-based diet negatively [35,36,37,38]. In contrast, little is known about the relationship between meat attachment and attitudes towards and consumption of plant-based meat alternatives. Previous research on plant-based meat alternatives has mainly focused on the relationship between consumer attitudes towards meat (MA—meat attachment) and their intention to reduce its consumption and increase the intake of alternative sources of plant protein [32,36,39]. The extent to which MA influences the propensity to choose plant-based alternatives was analyzed. Research has also examined how MA shapes consumers’ perceptions of the ideal sensory qualities of plant-based meat alternatives [40]. The relationship between consumers’ attachment to meat and concern for their health, attention to the naturalness of food, willingness to buy new foods, or willingness to try products from other countries has not been studied.

This study aimed to determine the relationship between meat attachment among Polish consumers and their willingness to change to the consumption of plant-based and meat alternatives, using a self-assessment including attitudes towards health, naturalness of food, and product novelty, and willingness to seek information about these products. In addition, the relationship between the meat attachment of people consuming plant-based meat alternatives and their attitudes towards these products was assessed.

## 2. Materials and Methods

### 2.1. Study Design and Sample Collection

A nationwide cross-sectional survey was conducted by the Research Agency between June and September 2023. A CAWI (computer-assisted web interview) method collected data from 1003 respondents. To ensure the representativeness of the sample and minimize potential sampling bias commonly associated with the CAWI methodology, a quota sampling approach was implemented. The quotas were based on national demographic data provided by Statistics Poland (GUS) and accounted for gender, age, and place of residence. The structure of the final sample was closely aligned with population benchmarks, increasing the reliability and generalizability of the findings. While quota sampling does not ensure full randomization, it allows for effective control of sample composition, particularly when used in combination with an online data collection tool. Confidentiality and anonymity of the data were ensured.

The following inclusion criteria were considered in the sample selection: gender (male and female), age above 18 years, eating meat at least once a week, and informed consent to participate in the study. Exclusion criteria were age below 18 years, eating meat less than once a week, and not agreeing to participate in the study.

This study was approved by the Ethics Committee of the Warsaw University of Life Sciences (Resolution No. 8/RKE//2023/U; 20 April 2023) and was conducted following the guidelines of the Declaration of Helsinki.

### 2.2. Questionnaire

The MAQ questionnaire was used to assess consumers’ attachment to meat consumption [36]. The MAQ has been used in various studies, for example, in an analysis of the motivations and attitudes of consumers from New Zealand towards meat consumption [35], to measure the meat paradox among the Australians [41], to research German consumer preferences for meat products and plant protein mixtures [42], and in a study of parents’ attachment to meat and limiting meat consumption among children [39,43]. This survey tool consists of 16 statements rated on a 5-point Likert scale from 0 (strongly disagree) to 4 (strongly agree). On our scale, 1 meant strongly disagree and 5 meant strongly agree.

The following questions assessed the intention to change the consumption behavior of plant-based foods and meat and meat products in the coming year: ‘Do you intend to eat more plant-based foods in the next year?’ (yes/no); ‘Do you intend to eat less meat and meat products in the next year?’ (yes/no). Information on the knowledge and consumption of plant-based meat alternatives was obtained by formulating the following questions: ‘Do you know and consume plant-based meat alternatives?’ (I do not know/I know, but I do not consume); ‘If you do not consume plant-based meat alternatives, do you intend to change this?’ (yes/no).

To measure the respondents’ opinions on plant-based meat alternatives, seven statements were used comparing these products with animal products (e.g., “Plant-based meat alternatives taste the same as animal products”), and these comparisons included taste, appearance, convenience of use, preparation time, nutritional value, health benefits, and price. The opinions were presented on a 5-point rating scale, where 1 was disagree and 5 was agree.

To measure self-identity, five self-descriptors were used, including attitudes towards one’s own health, the naturalness of food, new products, information about a new product, and consumption of products from different countries. An example of a statement regarding one’s own health is ‘I consider myself to be a person who cares about health’. A 5-point rating scale was used for each statement, where a rating of 1 means that the respondent does not agree with the statement, and a rating of 5 means that the respondent agrees.

Questions concerning the socio-demographic characteristics of the respondents took into account gender, age (in years), place of residence (village or town with less than 20,000 inhabitants, city with 20,000–100,000 inhabitants, city with 100,000–200,000 inhabitants, city with 200,000–500,000 inhabitants, city with over 500,000 inhabitants), level of education (primary, vocational, secondary, higher), and self-assessment of the financial situation (we have enough for everything without any special savings, we live frugally and we have enough for everything, we live very frugally to save for more serious purchases, we have enough money only for the cheapest food and clothes, we have enough money only for the cheapest food but not for clothes, we do not have enough money even for the most inexpensive food and clothes, I do not know/hard to say).

### 2.3. Statistical Analysis

Before starting the statistical analysis, the internal consistency of the individual subscales of the MAQ was determined using Cronbach’s alpha coefficient. The results (hedonism—0.90; affinity—0.85; entitlement—0.83; dependence—0.91) confirmed their satisfactory consistency. The scores for each subscale of the MAQ were then calculated by summing the scores for the statements making up the subscale, and the mean score for each scale was then calculated [34]. A higher score on a subscale indicates greater hedonism, entitlement, and dependence and lower affinity for meat.

As part of the statistical analysis, descriptive statistics were performed for the quantitative variables, and the distributions were analyzed for qualitative variables. The results calculated for the individual subscales of the MAQ were used to segment the study participants. Segmentation was performed using the k-means method, in which the mean values of the variables obtained in the hierarchical analysis performed earlier were used as the initial points of the clusters. The subjective evaluation regarding the number of clusters was supported by the CCC (cubic clustering criteria) statistic (25.36) and the pseudo F (550.13).

All the analyses were performed using the SAS 9.4 statistical package (SAS Institute, Cary, NC, USA) at a significance level of α = 0.05.

## 3. Results

### 3.1. Study Sample

The study sample consisted of 1003 adults aged 18–83. Women constituted more than one-half of the respondents. People with secondary education predominated (40.2%). Urban residents comprised 62.4%. Almost two-fifths of the respondents (37.6%) declared they lived frugally and had enough for everything. For 12.9% of the respondents, money was only enough for the cheapest food, and for 1.3%, money was not enough even for the most inexpensive food (Table 1).

### 3.2. Characteristics of the Identified Clusters

Based on the 16 variables belonging to the MAQ scale, four new variables were created: entitlement (mean value 3.7), hedonism (3.5), dependence (3.2), and affinity (2.3), which were used for segmentation. Four separate clusters were identified. Cluster 1, in contrast to the others, was characterized by higher scores of entitlement (4.8) and hedonism (4.7) and the lowest score for affinity (1.4). The respondents included in this cluster were called *Meat lovers* (Table 2). In cluster 2, the mean values for all the subscales adopted a similar level, and the respondents belonging to this cluster were called *Meat neutrals*. In cluster 3, as in cluster 1, the highest scores were obtained for entitlement (3.9) and hedonism (3.8) and the lowest for affinity (1.8), and these values were lower than in cluster 1. The respondents included in this cluster were called *Meat attached*. The opposite of Cluster 1 is Cluster 4, where the lowest levels of hedonism (1.8), entitlement (2.3), and dependence (1.9) and the highest level of affinity (3.0) were observed. The respondents representing cluster 4 were called *Meat opponents* (Table 2).

### 3.3. Socio-Demographic Characteristics and Declared Changes in Meat and Plant Food Consumption, Taking into Account Identified Clusters

Characteristics of the identified clusters with respect to gender, education level, and place of residence are presented in Table 3. No differences between the clusters were found with respect to age and self-reported financial situation.

In *Meat lovers*, compared to the other clusters, there were the fewest rural residents and the highest number of people from cities with 20,000 to 100,000 inhabitants, as well as the fewest people with higher education (26.8%) and the highest number of men (63.2%). There were more women than men among Meat-neutrals (57.8%). In addition, compared to other clusters, there were more respondents from towns with less than 20,000 inhabitants (16.4%) and people with vocational education (21.9%). The Meat-attached respondents were distinguished by equal proportions of men and women (49.3% and 50.7%, respectively). Meat opponents had the highest share of residents of the largest cities (18.3%), people with higher education (41.7%), and women (63.3%) compared to the other clusters.

The highest number of people declaring their intention to eat more plant-based foods but also to eat less meat and meat products in the next year were in Meat opponents (88.3% and 82.5%, respectively), followed by Meat-neutrals (74.1% and 60.2%, respectively). In contrast, Meat lovers were the least likely to declare such changes, with 31.0% declaring an increase in plant-based food consumption and only 9.1% declaring a reduction in meat and meat product consumption (Table 3).

### 3.4. Characteristics of the Identified Clusters with Regard to Self-Identity

The results describing the self-identity of the respondents are presented in Table 4. The mean values for the whole study group indicate that the respondents perceived themselves as caring about their health (3.7) and enjoying eating products from different countries (3.7). The respondents indicated that they were not afraid of consuming foods that were unfamiliar to them (2.8) and did not seek information about new foods (2.9). *Meat lovers* compared to *Meat opponents* represented people who cared more about their own health (3.9 vs. 3.6) but paid less attention to the naturalness of food products (3.5 vs. 3.7), liked to eat products from different countries more (3.9 vs. 3.6), and wanted to buy new food products (3.7 vs. 3.3). *Meat-neutral*, compared to the other clusters, were seeking more information about new food products (3.1) and, at the same time, were more concerned about eating unfamiliar food (3.0). *Meat-neutral* and *Meat opponents* were those who most often paid attention to the naturalness of food (3.6 and 3.7, respectively). It was found that the respondents representing *Meat opponents* did not like to buy new food products (3.3) and rarely sought information about new food products (2.7).

### 3.5. Perception of Plant-Based Meat Alternatives with Regard to Identified Clusters

**Figure 1:**
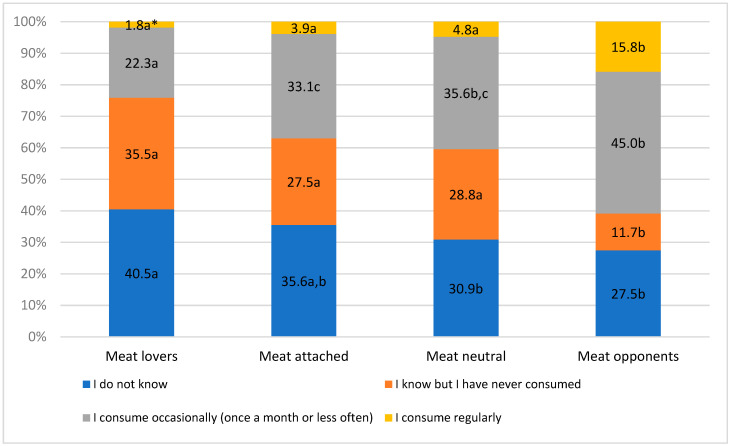
Familiarity with and consumption of plant-based meat alternatives, taking into account cluster affiliation. * Z-test—comparison of proportions; same letters indicate no statistically significant differences between clusters.

About one-third (33.9%) of the respondents were unfamiliar with plant-based meat alternatives. About 33.1% of respondents consumed them occasionally (once a month or less often), and 27.8% declared that they knew these products but had never consumed them. Only 5.2% of the surveyed respondents consumed plant-based meat alternatives regularly. *Meat lovers* and *Meat-attached* were most often unfamiliar with plant-based meat alternatives (40.5% and 35.6%, respectively). More respondents from *Meat lovers* and *Meat-neutrals* were familiar with them but never consumed them (35.5% and 28.8%, respectively). Most respondents from *Meat opponents* indicated that they consumed them occasionally and regularly (45.0% and 15.8%). At the same time, it was also noted that 27.0% of the study subjects representing *Meat opponents* were not familiar with plant-based meat alternatives (Figure 1).

Respondents consuming plant-based meat alternatives (384) agreed that, compared to animal products, plant-based meat alternatives were more expensive (3.7), more beneficial to health (3.6), and resembled animal products in appearance (3.5). The surveyed respondents tended to disagree with the statement that plant-based meat alternatives were products that had the same taste as meat products (2.7) (Table 5).

In *Meat opponents*, plant-based meat alternatives were more appreciated due to their beneficial effects on health (4.1), the convenience of use during food preparation (3.8), and appearance resembling animal products (3.6). Similarly to *Meat-neutral*, these respondents showed the highest level of agreement with the statement that plant-based meat alternatives had the same taste as animal products. *Meat lovers* did not perceive plant-based meat alternatives as products with the same taste (2.1) and nutritional value (2.6) as animal products. They were aware that these products were more expensive (3.6) and that they resembled animal products in their appearance (3.3). *Meat-attached* believed that plant-based meat alternatives were, above all, more expensive (3.9).

## 4. Discussion

The aim of this study was to determine the relationship between meat attachment among Polish consumers and their willingness to change their consumption of plant-based and meat alternatives, using a self-assessment, including attitudes towards health, naturalness of food, product novelty, and willingness to seek information about these products. In addition, the relationship between the meat attachment of people consuming plant-based meat alternatives and their attitudes towards these products was assessed.

The differentiation in meat attachment in the study group, resulting in the identification of four homogeneous clusters, namely, *Meat lovers*, *Meat-neutral*, *Meat-attached*, and *Meat opponents*, is confirmed by previous studies [44] and is largely determined by cultural and culinary conditions [9,45,46] but also by psychosocial factors, including attitudes towards meat and plant-based meat substitutes [47,48].

The attachment to meat in Polish society, as in many other countries, results from the fact that meat has always played an important role [49,50,51]. Poland has remained one of the leaders in meat consumption in Europe for years. In 2023, each resident of Poland consumed an average of 77.8 kg of meat. The most consumed meat was poultry (31.5 kg per person) and pork (40.5 kg per person). Beef consumption fluctuated at 0.8 kg per person [52]. A 2024 report by the Institute of Public Affairs titled “Will the Polish consumer support the transformation of the food system?” shows that the reduction in meat consumption is slow. It found that 87% of Poles remain on a classic diet—eating whatever their health allows [44]. In many cultures, meat has been and continues to be associated with wealth, power, and masculinity [53]. However, changes in meat consumption are currently being observed, with an increasing number of people limiting their meat consumption or excluding meat from their diet [54,55], which may be due to a lower attachment to meat, as confirmed not only by the *Meat opponents* cluster but also by *Meat-neutrals*.

In addition to cultural determinants, the diversity of attitudes towards meat and consumers’ relationships with the willingness to limit its consumption has been demonstrated in many studies [56,57]. The application of the Theory of Planned Behavior [35,58,59,60] and the Health Belief Model [61,62] in explaining the intention to restrict meat consumption confirms the significance of attitudes towards meat and, thus, attachment to meat.

These results can also be interpreted in light of established theoretical models of behavior, particularly the Theory of Planned Behavior [58] and the Health Belief Model [61]. According to the TPB, attitudes toward behavior, subjective norms, and perceived behavioral control determine intentions to perform behavior.

The health belief model similarly provides insight into the role of perceived health benefits and perceived barriers in shaping dietary choices. Respondents who value health and are open to plant-based foods may be motivated by perceived benefits, such as disease prevention, as well as social or informational cues.

The results of our study show that more than three-fifths of the respondents (60.4%) were willing to increase their consumption of plant products, but only slightly fewer people did not intend to reduce their meat consumption (57.9%). Many other populations also report a reluctance to reduce meat consumption [35,56,57]. The lack of planned changes in meat consumption in the study group is consistent with the results of the report of the Institute of Public Affairs, indicating that the reduction in meat consumption in Poland is slow. It was found that 87% of Poles follow a classic diet, 4% of the respondents did not eat meat, and another 7% limited its consumption [63].

The observed patterns in our study are in line with research conducted in Germany [42], The Netherlands [64], and New Zealand [35] which similarly emphasize health concerns, ethical beliefs, and perceived sensory qualities as key drivers of reduced meat consumption and increased openness to alternatives. However, our findings contrast with reports from countries with longer exposure to PBMA, where regular consumption is more common. The relatively low familiarity and consumption rates observed in the study sample may reflect cultural norms, lower market penetration, or lack of product trust.

The willingness to increase the consumption of plant-based foods while maintaining meat consumption (declared lack of intention to reduce its consumption) at the current level confirms the direction of changes reflecting the idea of “flexitarianism”, characterized by combining the consumption of meat products with a greater number of plant-based alternatives [65]. Such changes help maintain a balance between the health benefits of a more plant-based diet and the desire to preserve dietary traditions [55].

Meat attachment differentiated declarations regarding changes in meat and plant-based food consumption. The smallest number of *Meat lovers* declared their intention to reduce meat consumption and simultaneously increase their consumption of plant-based food in the next year, which indicates that changing to a more plant-based diet may be a challenge for meat-loving individuals [64,66,67]. The respondents belonging to this cluster were distinguished by the highest scores for ‘entitlement’ and ‘hedonism’; thus, eating meat is a source of pleasure and reward for them [19]. Men predominated in this cluster (63.2%), confirming their strong attachment to meat as the main component of their daily diet [33,53,68]. In turn, *Meat opponents*, characterized by the lowest attachment to meat, declared the greatest willingness to increase the consumption of plant products and reduce meat consumption in the next year. The *Meat opponents* were dominated by women (63.3%), whose greater interest in a plant-based diet was confirmed in many previous studies [40,69,70,71]. Apart from gender, the respondents’ education and place of residence also showed differences in both clusters. The *Meat-lovers* cluster was dominated by respondents with lower education, while the Meat-opponents cluster had the largest number of people with higher education. Previous studies have shown that the latter are more likely to make informed dietary choices [72,73], which may explain the differences in declarations related to meat and plant food consumption. The *Meat lovers* were mostly residents of cities with 20,000 to 100,000 inhabitants, while the *Meat opponents* were mostly residents of cities with more than 500,000 inhabitants. In rural areas, there were few *Meat lovers* and *Meat opponents*. Meat consumption is still common in rural areas [74], and at the same time, changes are observed in this regard [75]. However, cultural norms and expectations regarding eating meat [74,76] promote attachment to meat, and thus may slow down changes in its consumption. In addition, the cultural attachment to meat in the rural environment may be accompanied by limited access to plant foods due to long distances to shops or financial barriers [77,78].

The results indicate that attachment to meat differentiates the familiarity with and frequency of consumption of plant-based meat alternatives. Although these products are becoming increasingly important due to the environmental, social, and ethical challenges posed by the production and consumption of animal products [66,79,80], in the study group, only about 5% of the respondents consumed them regularly, and 33.9% never consumed them. This may be due to their relatively short availability on the market. In Poland, this market has been developing only since around 2015. In Western societies, these products are still a novelty that has a low level of acceptance by consumers [81,82]. In the case of the *Meat lovers*, 74% of the respondents were not familiar with and did not consume plant-based meat alternatives, while almost 70% of Meat opponents consumed plant-based meat alternatives occasionally. The occasional consumption of plant-based meat alternatives dedicated to consumers who wish to consume less meat [15] may result from existing structural barriers [66]. Limited availability, being relatively new, and being perceived as expensive [83] may be a barrier to their consumption in the *Meat opponents*, as well. On the other hand, for other people, motivational barriers may be of great importance in limiting their consumption [66], including food neophobia [40,71,84,85] and local food norms and customs [86], as well as conflicting eating goals [66].

Plant-based meat alternatives were perceived as expensive products, especially among *Meat-attached*. High price was also a significant barrier to choosing these products in other studies [71,87]. *Meat opponents* were less likely to report a higher price for plant-based meat alternatives than for animal products, and at the same time, they were more convinced that these products have the same nutritional value as animal products. Previous studies have shown that consumers perceive plant-based meat alternatives as products that have a positive impact on health [88]. However, the beneficial effects of these products on health were more noticed by *Meat opponents*, while *Meat lovers* were the most skeptical on this issue.

Nowadays, the lack of acceptance of the taste leads to a refusal to consume the product again [89]. Hence, plant-based meat alternatives are being developed to imitate the sensory characteristics of meat to increase their acceptability [82,90,91]. In the study group, plant-based meat alternatives were not perceived as products with the same taste as animal products (average value 2.7), which is also confirmed by other studies [92,93,94]. The lack of comparability of taste of both product groups was declared mainly by *Meat lovers*, while *Meat-neutrals* noticed such similarity the most. In addition to the taste of plant-based meat alternatives, other features of these products are also important for consumers, including appearance and consistency [85,95,96]. Within the study group, *Meat lovers* were least likely to agree with the statement that plant-based meat alternatives resemble animal products in appearance, which, together with the opinion that they are not comparable in terms of taste, may explain the lowest consumption of these products among those most attached to meat. *Meat opponents*, on the other hand, were more likely to express the opinion that the use of plant-based meat alternatives reduces the time that needs to be spent on food preparation. In addition, they felt that compared with meat, plant-based meat alternatives are more convenient when preparing dishes at home. These positive opinions are further supported by the belief that they can be prepared for consumption similarly to meat [92,97,98,99], accounting for the higher consumption of plant-based meat alternatives in the *Meat opponents*.

The respondents belonging to the identified clusters showed variation due to some socio-demographic characteristics (gender, education, place of residence) but also due to self-perception. It turned out that *Meat opponents* perceived themselves as not afraid of unfamiliar foods, as indicated by the lowest score for fear of eating foods they had never eaten (mean value 2.4). On the other hand, they did not see themselves as innovators, as indicated by the low score for liking to buy new products (mean value 3.3). This may mean that they are not afraid to try unfamiliar foods but need some time to do so, so exposure to new foods becomes important in their situation. The *Meat lovers*’ declarations indicate that they are the most innovative, as they like buying new foods and consuming products from other countries. At the same time, the fewest of these people consumed plant-based meat alternatives, which, when considering plant-based meat alternatives as market novelties, calls for further research explaining the relationship between meat attachment, innovation, and behavior. *Meat opponents*, on the other hand, valued the naturalness of the food. Given that the consumption of plant-based meat alternatives was highest in this group, the question arises as to whether the high importance of naturalness of food should not be a barrier to their acceptance, as most of them fall into the category of ultra-processed foods [100]. The production of minimally processed, clean-label plant-based meat alternatives could further increase the interest in purchasing such food among people who value natural food [101].

Our study shows that neither *Meat lovers* nor *Meat opponents* were interested in seeking information about new food products, which include plant-based meat alternatives. Such activities were most popular among people representing the *Meat-neutral* cluster. This diversity of the need for information should pose a challenge for people involved in educational and marketing activities aimed at promoting the health and environmental benefits associated with reducing meat consumption in favor of plant-based foods. Increasing interest in plant-based meat alternatives also requires taking into account differences in consumers’ willingness to seek information about them. It is also crucial to reduce barriers perceived by consumers, such as high prices, unacceptable taste, and low nutritional value of plant-based meat alternatives.

## 5. Strengths and Limitations of This Study

The strength of this study is its approach to using food attachment in explaining changes in dietary behavior. The identification of homogeneous clusters concerning attachment to meat allows for a more comprehensive look at the possible directions of changes in the consumption of plant and animal food and their barriers.

This study has some limitations that should be mentioned. A limitation of our study is that although the sample was representative in terms of gender, age, and place of residence, specific cultural groups or regional differences in Poland that may influence food preferences were not taken into account. This study was conducted exclusively using the CAWI (computer-assisted web interview) method, which limits access to respondents who do not actively use the Internet. This may lead to underrepresentation of age groups, such as elderly people, who may have different attitudes towards meat and plant-based meat alternatives. In addition, this study concerned only Polish consumers, which limits the possibility of generalizing the results to other countries with different culinary traditions and attitudes towards meat.

Another limitation of this study is also the lack of in-depth psychographic analysis, including the respondents’ values, beliefs, or lifestyles, which may influence dietary choices.

Another limitation relates to the use of the Meat Attachment Questionnaire (MAQ) as the primary instrument for segmentation. While MAQ is a validated tool with high internal consistency, it does not account for broader factors influencing dietary behavior, such as environmental concerns, identity-related symbolism, or emotional ambivalence.

Furthermore, this study relied entirely on self-reported data, which is inherently vulnerable to certain biases.

An additional limitation of this study is its cross-sectional design, which does not allow for causal inferences. While significant associations were observed between meat attachment, attitudes, and intentions to change dietary behaviors, the direction of these relationships remains unclear. Future studies using longitudinal or experimental designs are needed to explore these mechanisms in greater depth.

## 6. Conclusions

The survey found that the majority of respondents (60.4%) declared a willingness to increase their consumption of plant-based products, but a significant proportion (57.9%) did not intend to reduce meat consumption. Consumer attitudes towards meat and its plant-based alternatives varied, allowing four clusters to be identified: *Meat lovers, Meat-neutral, Meat-attached* and *Meat opponents*.

*Meat lovers* showed the strongest attachment to meat, while *Meat opponents* were more likely to consume plant-based meat alternatives, appreciating their naturalness and health benefits. Knowledge and consumption of plant-based meat alternatives in the study group was low. In addition, these products were perceived as expensive, with less appealing taste and lower nutritional value compared to traditional meat products, which was particularly evident in the *Meat lovers* group.

In order to increase the consumption of plant-based foods, including plant-based meat alternatives, and thus reduce meat consumption, targeted education and marketing activities are needed. These should highlight the health and environmental benefits of reducing meat consumption. It is crucial to tailor the message to different groups of consumers, taking into account motivations such as the need to explore new flavors, sensitivity to health issues, and the naturalness of products.

## Figures and Tables

**Table 1:** Characteristics of study sample.

	Total	
	N *	%
Total	1003	100.0
Gender		
Men	483	48.2
Women	520	51.8
Age (years)		
18–24	104	10.4
25–34	193	19.2
35–44	205	20.4
45–54	162	16.2
55–64	221	22.0
65 and above	118	11.8
Education		
Primary	100	10.0
Vocational	180	17.9
Secondary	403	40.2
Higher	320	31.9
Place of residence before studying		
A village	377	37.6
A town with less than 20,000 inhabitants	139	13.9
A city with 20,000–100,000 inhabitants	187	18.6
A city with 100,00–200,000 inhabitants	104	10.4
A city with 200,000–500,000 inhabitants	93	9.3
A city with over 500,000 inhabitants	103	10.3
Self-reported financial situation We have enough for everything without any special savings	170	16.9
We live frugally and we have enough money for everything	377	37.6
We live very frugally to save for major purchases	269	26.8
We have only enough money for the cheapest food and clothes	88	8.8
We have enough money only for the cheapest food but not for clothes	41	4.1
We do not have enough money even for the most inexpensive food and clothes	13	1.3
I do not know/hard to say	45	4.5
Age (mean; SD in years) (range)	45.4; 15.55 (18–83)	

* N—number of participants.

**Table 2:** Mean values of variables for meat attachment.

		Total (N = 1003)	Cluster 1 (N = 220)	Cluster 2 (N = 379)	Cluster 3 (N = 284)	Cluster 4 (N = 120)	*p*
Meat Attachment	Hedonism	3.5 (0.98) *	4.7 a (0.37) **	3.1 c (0.54)	3.8 b (0.47)	1.8 d (0.60)	<0001
	Entitlement	3.7 (0.93)	4.8 a (0.34)	3.3 c (0.56)	3.9 b (0.46)	2.3 d (0.79)	<0001
	Affinity	2.3 (0.95)	1.4 c (0.62)	2.9 a (0.61)	1.8 b (0.54)	3.0 a (0.98)	<0001
	Dependence	3.2 (0.77)	4.1 a (0.36)	3.0 c (0.53)	3.3 b (0.47)	1.9 d (0.44)	<0001

* Mean value (standard deviation) obtained from a 5-point scale, where 1 is disagree; 2 is rather disagree; 3 is neither agree nor disagree; 4 is rather agree; and 5 is agree; ** Comparison of means between clusters—ANOVA test with Wallis post hoc test. Different letters at the means in a row indicate significant differences between clusters (*p* < 0.05).

**Table 3:** Socio-demographic characteristics and declared changes in meat and plant food consumption, taking into account identified clusters.

Items		Total Sample (N = 1003)	C1 Meat Lovers (N = 220)	C2 Meat-Neutral (N = 379)	C3 Meat-Attached (N = 284)	C4 Meat Opponents (N = 120)	*p* *
Gender	Men Women	48.2 51.8	63.2 36.8	42.2 57.8	49.3 50.7	36.7 63.3	<0.001
Education	Primary Vocational Secondary Higher	10.0 17.9 40.2 31.9	12.3 16.4 44.5 26.8	9.0 21.9 36.9 32.2	8.5 15.1 45.1 31.3	12.5 15.0 30.8 41.7	0.017
Place of residence	A village A town with less than 20,000 inhabitants A city with 20,000–100,000 inhabitants A city with 100,00–200,000 inhabitants A city with 200,000–500,000 inhabitants A city with over 500,000 inhabitants	37.6 13.9 18.6 10.4 9.3 10.3	30.5 14.1 25.5 11.4 10.5 8.2	40.4 16.4 15.3 10.5 9.0 8.4	40.5 12.7 18.0 8.8 9.1 10.9	35.0 8.3 18.3 11.7 8.3 18.3	0.018
Intention to eat more plant-based foods in the next year	Yes No	60.4 39.6	31.8 68.2	74.1 25.9	52.5 47.5	88.3 11.7	<0.001
Intention to eat less meat and meat products in the next year	Yes No	42.1 57.9	9.1 90.9	60.2 39.8	26.4 73.6	82.5 17.5	<0.001

* Chi-square test; significance at *p* < 0.05.

**Table 4:** Characteristics of clusters in terms of self-identity.

I Consider Myself to Be a Person Who	Total Sample (N = 1003)	C1 Meat Lovers (N = 220)	C2 Meat Neutral (N = 379)	C3 Meat Attached (N = 284)	C4 Meat Opponents (N = 120)	*p*
Cares about my health	3.7 (0.94) *	3.9 a ** (0.97)	3.7 ab (0.87)	3.7 ab (0.87)	3.6 b (1.20)	0.032
Draws attention to the naturalness of food	3.6 (1.00)	3.5 b (1.19)	3.6 ab (0.84)	3.5 ab (0.97)	3.7 a (1.12)	0.037
Likes to buy new food products	3.5 (1.06)	3.7 a (1.18)	3.4 b (0.96)	3.6 ab (0.97)	3.3 b (1.27)	0.019
Seeks information about new food products	2.9 (1.18)	2.8 b (1.32)	3.1 a (1.04)	2.9 b (1.17)	2.7 b (1.24)	0.000
Likes to eat products from different countries	3.7 (1.06)	3.9 a (1.18)	3.6 b (0.92)	3.7 b (1.04)	3.6 b (1.23)	0.005
Is afraid to eat foods I have never tried	2.8 (1.15)	2.8 ab (1.34)	3.0 a (0.97)	2.7 b (1.10)	2.4 c (1.30)	<0001

* Mean value (standard deviation) from a 5-point scale, where 1 is disagree; 2 is rather disagree; 3 is neither agree nor disagree; 4 is rather agree; and 5 is agree; ** Comparison of means between clusters—ANOVA test with Wallis post hoc test. Different letters at the means in a row indicate significant differences between clusters (*p* < 0.05).

**Table 5:** Consumers’ opinions on the characteristics of plant-based meat alternatives.

Plant-Based Meat Alternatives	People Who Consume Plant-Based Meat Alternatives (N = 384)	C1 Meat Lovers (N = 53)	C2 Meat-Neutral (N = 153)	C3 Meat-Attached (N = 105)	C4 Meat Opponents (N = 73)	*p*
Taste the same as animal products	2.7 (1.14) *	2.1 c ** (1.27)	3.0 a (0.98)	2.5 b (1.09)	2.7 ab (1.24)	<0.0001
Resemble animal products in appearance	3.5 (0.96)	3.3 a (1.23)	3.6 a (0.82)	3.5 a (0.87)	3.6 a (1.12)	0.041
Have the same nutritional value as animal products	3.2 (1.07)	2.6 b (1.25)	3.4 a (0.95)	2.9 b (0.95)	3.6 a (1.07)	<0.0001
Are more expensive than animal products	3.7 (1.06)	3.6 ab (1.23)	3.6 ab (0.98)	3.9 a (0.98)	3.5 b (1.14)	0.0302
Are more convenient to use when I am preparing a dish at home	3.5 (0.96)	3.0 c (1.17)	3.6 b (0.87)	3.4 b (0.84)	3.8 a (0.98)	<0.0001
Reduce the time I have to spend preparing a dish	3.2 (1.02)	2.8 a (1.14)	3.3 ab (0.94)	3.1 bc (0.87)	3.5 a (1.15)	0.0003
Are beneficial to me for health reasons	3.7 (1.00)	2.9 d (1.28)	3.8 b (0.86)	3.5 c (0.83)	4.1 a (0.93)	<0.0001

* Mean value (standard deviation) from a 5-point scale, where 1 is disagree; 2 is rather disagree; 3 is neither agree nor disagree; 4 is rather agree; and 5 is agree. ** Comparison of means between clusters—ANOVA test with Wallis post hoc test. Different letters at the means in a row indicate significant differences between clusters (*p* < 0.05).

## Data Availability

The original contributions presented in the study are included in the article, further inquiries can be directed to the corresponding author.
